# An LCMS-based untargeted metabolomics protocol for cochlear perilymph: highlighting metabolic effects of hydrogen gas on the inner ear of noise exposed Guinea pigs

**DOI:** 10.1007/s11306-019-1595-1

**Published:** 2019-10-05

**Authors:** Kristian Pirttilä, Pernilla Videhult Pierre, Jakob Haglöf, Mikael Engskog, Mikael Hedeland, Göran Laurell, Torbjörn Arvidsson, Curt Pettersson

**Affiliations:** 1 Department of Medicinal Chemistry, Uppsala University, Uppsala, Sweden; 2 Division of Audiology, Department of Clinical Science, Intervention and Technology, Karolinska Institutet, Stockholm, Sweden; 3 Department of Surgical Science, Uppsala University, Uppsala, Sweden

**Keywords:** Metabolomics, NIHL, In vivo, Noise-induced hearing loss, LCMS, Perilymph

## Abstract

**Introduction:**

Noise-induced hearing loss (NIHL) is an increasing problem in society and accounts for a third of all cases of acquired hearing loss. NIHL is caused by formation of reactive oxygen species (ROS) in the cochlea causing oxidative stress. Hydrogen gas (H_2_) can alleviate the damage caused by oxidative stress and can be easily administered through inhalation.

**Objectives:**

To present a protocol for untargeted metabolomics of guinea pig perilymph and investigate the effect of H_2_ administration on the perilymph metabolome of noise exposed guinea pigs.

**Methods:**

The left ear of guinea pigs were exposed to hazardous impulse noise only (*Noise*, n = 10), noise and H_2_ (*Noise *+ *H2*, n = 10), only H_2_ (*H2*, n = 4), or untreated (*Control*, n = 2). Scala tympani perilymph was sampled from the cochlea of both ears. The polar component of the perilymph metabolome was analyzed using a HILIC-UHPLC-Q-TOF–MS-based untargeted metabolomics protocol. Multivariate data analysis (MVDA) was performed separately for the exposed- and unexposed ear.

**Results:**

MVDA allowed separation of groups *Noise* and *Noise *+ *H2* in both the exposed and unexposed ear and yielded 15 metabolites with differentiating relative abundances. Seven were found in both exposed and unexposed ear data and included two osmoprotectants. Eight metabolites were unique to the unexposed ear and included a number of short-chain acylcarnitines.

**Conclusions:**

A HILIC-UHPLC-Q-TOF–MS-based protocol for untargeted metabolomics of perilymph is presented and shown to be fit-for-purpose. We found a clear difference in the perilymph metabolome of noise exposed guinea pigs with and without H_2_ treatment.

**Electronic supplementary material:**

The online version of this article (10.1007/s11306-019-1595-1) contains supplementary material, which is available to authorized users.

## Introduction

Noise induced hearing loss (NIHL) is a common disorder that accounts for a third of all cases of acquired hearing loss worldwide (Le et al. 2017; Sha and Schacht 2017). The cochlea is a structure of the inner ear that harbor the sensory cells of hearing and is filled with endolymph and perilymph. Noise exposure can lead to pathological changes in the cochlea causing hearing impairment. One key mechanism is the formation of reactive oxygen species (ROS) leading to oxidative stress in the inner ear (Henderson et al. 2006; Le et al. 2017). The ROS may leak into the surrounding tissue, causing widespread damage over the course of several days (Le et al. 2017). In the past, hydrogen gas (H_2_) has been shown to reduce oxidative stress-induced effects in a number of organ systems (Fukuda et al. 2007; Huang et al. 2011). In addition, H_2_ has been shown to reduce hearing loss due to noise exposure (Lin et al. 2011; Kurioka et al. 2014) and cisplatin ototoxicity (Qu et al. 2012; Kikkawa et al. 2014; Fransson et al. 2017). However, more knowledge about the molecular mechanisms surrounding the protective effect is needed.

Blood is a common sample material in metabolomics studies owing to the relative ease of sampling. As blood permeates the entire body it comes in contact with every organ. Subsequently, the blood metabolite composition represents an approximate average of the metabolic states of the whole body (Psychogios et al. 2011). When studying inherently localized biological effects the use of localized tissue or fluids sampled in proximity to the afflicted region is preferable as these are more representative of the system being studied. In that respect, cochlear perilymph is in close contact with the sensory cells of hearing, the inner and outer hair cells, making it highly suitable for studying acquired hearing loss. However, perilymph sampling cannot be performed in humans without causing iatrogenic hearing loss. It is also challenging in experimental animals since only a very small volume can be drawn to avoid the risk of contamination with cerebrospinal fluid (CSF) (Hara et al. 1989). In this work, *scala tympani* perilymph from guinea pigs, a frequently used species in hearing research, was analyzed as this can be safely sampled up to 1 µL using available surgical techniques. As a consequence of the difficulty in obtaining samples, perilymph is a relatively unknown sample material in terms of bioanalysis. Most of the literature on the composition of human perilymph has been focused on its proteome content and is limited to patients undergoing surgical interventions (Lysaght et al. 2011; Schmitt et al. 2017; Edvardsson Rasmussen et al. 2018; Palmer et al. 2018). Some attention has been directed at characterizing the small metabolites of perilymph using mainly HPLC with fluorescence detection (Drescher, Medina and Drescher 1981; Medina and Drescher 1981; Scheibe and Haupt 1985; Bobbin and Fallon 1992). The qualitative composition of perilymph, in terms of proteins and small metabolites, is comparable to that of both CSF and blood plasma, whereas the relative concentrations differ widely between the three fluids (Scheibe and Haupt 1985; Leary Swan et al. 2009; Fujita et al. 2015). Notably, Fujita et al. (2015) used a GCMS-based metabolomics protocol to study the effect of noise on the metabolome of the inner ear fluid of guinea pigs. They found ten metabolites that were significantly altered in concentration in inner ear fluid, whereas no altered metabolite concentrations were found in blood. Recently, Mavel et al. (2018) used LCMS to characterize the metabolites of human perilymph in patients undergoing cochlear implantation. Most likely due to the limited availability of perilymph from healthy subjects, no control samples were included in their study, and no group comparison was made. However, they were able to identify a large number of compounds from various biological pathways using their developed protocol.

LCMS, in contrast to GCMS, allows the detection of thermally labile and highly polar compounds without need of derivatisation. To the best of our knowledge, untargeted metabolomics using LCMS has not previously been applied to study hearing loss. Furthermore, the use of H_2_ to attenuate NIHL has not been studied at the metabolite level. To that end, we present an LCMS-based untargeted metabolomics protocol for perilymph applied to a guinea pig model of the attenuating effect of H_2_ on NIHL.

## Materials and methods

### Chemicals

All water was purified using a Milli-Q™ water system from MilliPore (Bedford, MA, USA). Ammonium formate (LCMS-grade) and formic acid (LCMS-grade) were obtained from Sigma-Aldrich (Steinheim, Germany). Acetonitrile (LCMS-grade) was purchased from Fisher Scientific (Zurich, Switzerland). Acetonitrile (ACN) used for protein precipitation was kept on ice throughout the sample preparation procedure. Butyryl-l-carnitine, creatine, *O*-acetyl-l-carnitine hydrochloride, and stachydrine-(dimethyl-^13^C_2_) monohydrate were obtained from Sigma-Aldrich.

### Software

LCMS equipment was controlled using MassLynx (ver. 4.1, Waters Corp., Milford, USA). Databridge (ver. 3.5, Micromass UK Ltd., Manchester, England) was used to convert .raw files to .cdf files. All data preprocessing and univariate analysis were performed in the R statistical language environment (ver. 3.4.2). Peak picking, feature detection, retention alignment, and peak filling were performed using the XCMS R-package (Smith et al. 2006). The IPO R package (Libiseller et al. 2015) was used to find pseudo-optimal XCMS parameters. Adduct annotation was performed using the CAMERA R-package (Kuhl et al. 2012; Kuhl et al. 2014). Multivariate data analysis (MVDA) was performed in SIMCA (ver. 14.1, Umetrics AB, Umeå, Sweden).

### In vivo procedures

All animal procedures and results of auditory brainstem response (ABR) assessment and histology are reported in full elsewhere (Videhult Pierre et al. Hydrogen gas inhalation attenuates acute noise trauma. A preclinical in vivo study, *in manuscript*). In short, the left ear of guinea pigs was exposed to hazardous impulse noise (156 dB, 400 impulses, ~ 4 min) only (*Noise*; n = 10) immediately followed by inhalation of H_2_ (2% in air, 60 min; *Noise *+ *H*_*2*_; n = 10). Additional animals received only H_2_ (*H2*; n = 4) or no treatment at all (*Control*; n = 2). After ABR recordings at day 4, 1 µL of *scala tympani* perilymph was sampled using calibrated capillary tubes from the basal turn of each cochlea (Hellberg et al. 2013) added to a pre-chilled vial containing 19.0 µL H_2_O, and stored at − 80 °C until further treatment.

### Sample preparation

Sample preparation was performed in batches of 30 samples. Batches were randomized to achieve an even distribution of sample groups across all batches. Protein precipitation was performed on ice by addition of 20.0 µL ice-cold acetonitrile (4:1, v/v) to 5.0 µL sample aliquots. A quality control (QC) sample was prepared as a pooled mixture of sample aliquots and subjected to the same protein precipitation protocol. Samples were precipitated at 8 °C for 30 min and centrifuged (21,000×*g*, 4 °C, 15 min). The supernatant was kept at − 80 °C for no more than 7 days. Before analysis all samples were analyzed without further treatment. Samples were kept at 4 °C throughout the analytical run.

### LC-ESI-Q-TOF MS sample analysis

Chromatographic separation and detection was achieved using an ACQUITY I-class UPLC system (Waters Corp., Milford, USA) coupled to a Synapt G2-S QTOF HRMS instrument (Waters).

#### Chromatographic separation

Chromatographic separation was performed in HILIC mode using a Waters BEH Amide column (50 × 2.1 mm i.d., 1.7 µm particle size, 100 Å pore size) fitted with a Waters VanGuard BEH Amide (5 × 2.1 mm i.d., 1.7 µm particle size, 100 Å pore size) pre-column. Mobile phase A was prepared by addition of 100 mL ammonium formate buffer (200 mM in water, adjusted to pH 3 using formic acid) to 1900 mL ACN. Mobile phase B was prepared by addition of 100 mL ammonium formate buffer (200 mM in water, pH 3) and 100 mL ACN to 1800 mL water. Strong- and weak wash solutions were 10:90 and 95:5 ACN:water (v/v), respectively. All mixtures were degassed by ultrasonication for 20 min. Elution was performed using a gradient from 0 to 61%B over 14 min using a non-linear concave gradient (setting 8 in MassLynx, see supporting info for a definition) and kept at 61%B for 2.7 min followed by a gradient back to 0%B over 0.3 min and re-equilibration at 0%B for 6 min. The column temperature was 40 °C. Sample injection volume was 4 µL.

#### MS detection

Data were collected in ESI+ and ESI− mode in two different analytical runs. Mass calibration was performed according to manufacturer guidelines using sodium formate. Centroided mass data were acquired in resolution mode. For a full list of tune settings, see Supplementary Material. Internal mass calibration was achieved using a solution of leucine-enkephalin (0.2 nM in 1:1 ACN:water + 0.1% formic acid) infused at 10 µL/min and sampled every 15 s with a scan time of 0.3 s. Data was acquired in MS^E^ mode consisting of a full scan step between *m/z* 50–800 with a 1.0 s scan time followed by a high energy scan between *m/z* 50–800 with a 1.0 s scan time and transfer collision energy ramp from 20 to 45 eV.

### Quality control

#### Pre-analytical quality control

System suitability was assessed using an internal protocol and comparison to historic data for the instrument. In short, a solution of paracetamol, leucine-enkephaline, and meloxicam was injected and the result plotted as response intensity and mass accuracy as a function of time.

Prior to starting the analytical run the instrument was conditioned by repeated injection of the QC sample (Sangster et al. 2006; Gika et al. 2007). Stability was assessed in two ways: using (i) a targeted protocol of a set of known endogenous compounds detected in the QC sample, and (ii) an untargeted protocol using PCA of data generated from the conditioning injections using MarkerLynx (Waters). The drift in retention times and peak intensities were monitored until the RSD of peak intensities and retention times was < 10% and < 1%, respectively across five consecutive injections. For a full list of substances used for monitoring the conditioning, see Supplementary Material. Principal component analysis (PCA) was performed successively and the scores plot monitored until convergence of the points in the first and second component.

#### Inter-analytical quality control

The analytical run was initiated by injecting the QC sample. The analytical run then consisted of seven sample injections followed by a QC sample injection until all samples had been analyzed. Throughout the analytical run the same set of metabolites used to monitor the conditioning step was monitored in the QC sample injections. Retention time and peak intensity were determined to assess the presence of any sudden changes or major drift in instrument performance during the analytical run.

#### Post-analytical quality control

To assess the residual systematic drift and instrumental noise following data pretreatment, the full dataset including the QC sample injections was analyzed using PCA. This allows the existence and relative scale of such variation to be assessed in relation to the biological variation between samples as well as the detection of potentially outlying samples or features. Systematic error in peak intensities originating from instrument performance drift was assessed from the scores plot by the relative spatial distribution of the QC sample injections in relation to the distribution of study samples. The QC sample injections should cluster tightly at the center of the study samples.

### Data preprocessing

XCMS parameters were optimized using the IPO package and adjusted to be slightly more inclusive. CAMERA was used for adduct annotation using an extended ruleset (Stanstrup et al. 2013). See Supplementary Information for the initial parameters used with IPO, the final pseudo-optimized XCMS parameters, and the CAMERA parameters. Peak intensity normalization was performed by calculating the average feature intensity across all QC sample injections and calculating the feature-wise fold change of every sample to this average QC sample injection. Each sample was then normalized by dividing each feature intensity with the median of all feature-wise fold changes to the average QC sample injection (Dieterle et al. 2006; Veselkov et al. 2011; Wu and Li 2016). Features with a retention time of 60–800 s and a peak area RSD < 30% in the QC sample injections were kept.

### Multivariate data analysis and feature selection

PCA was performed on pareto-scaled data and was used to detect potential outliers, assess data quality, and visualize major structures in the data. Orthogonal projection to latent structures discriminant analysis (OPLS-DA) was used to model group membership in samples to find features important to group classification. The models used for feature selection was fitted on the full data set using 7-fold cross validation and overfit was assessed based on the PCA analysis, R2 and Q2 metrics, permutation tests (n = 500), as well as CV-ANOVA (Eriksson, Trygg and Wold 2008) in SIMCA. The classification error was estimated by leave-one-out cross validation (LOOCV) using 7-fold cross validation to fit models to the training sets and is further described in the Supporting Information. The correct classification rate (CCR) was determined as the ratio of the correctly classified samples over the total number of samples. Feature selection and comparison of models fitted on data from the left- and right ear was performed using the S- and SUS-plot (Wiklund et al. 2008) in SIMCA. A feature was selected for further study if it had a |p(corr)| > 0.5 or belonged to a cluster centered above this value. Adducts and fragments with comparable |p(corr)|-values were also included in the selection based on adduct annotation from the CAMERA package.

### Peak verification of selected features

Each feature selected in the MVDA step was subjected to manual inspection of the raw data in terms of peak shape and signal-to-noise (S/N) ratio. A peak was considered valid if it had a S/N over 10, an approximate Gaussian peak shape and no disturbing isobaric coelutions or irregularities in the peak that could make integration highly variable. Furthermore, adducts and fragments were manually verified by comparison of peak shape and retention times, grouping features with overlapping peaks and m/z differences corresponding to adducts and known fragments. Each peak was also cross-referenced with the original dataset to verify that it was not the only adduct being indicated by the model. In such cases, the feature was considered an artefact and was excluded.

For univariate analysis, the raw data was integrated using the TargetLynx module of MassLynx (Waters) and the peak integrals of each sample were normalized using the same normalization factor as in the data-preprocessing of the whole dataset.

### Univariate data analysis of selected features

Significance testing of important features was performed using the Wilcoxon two-sided rank-sum test. Visualization of the relative peak areas of each sample group was done using box plots.

## Results and discussion

### Quality control of LCMS analysis

#### Pre-analytical quality control

The system suitability test described above showed that no clear systematic deviation was observed in relation to historical instrument response over time and it was concluded that the instrument was performing nominally.

In the conditioning step, a stable system should show convergence of retention times and peak areas in the targeted approach as well as convergence to a cluster in the first two components of the PCA scores plot. Prior to starting the analytical run, in positive mode the peak area RSD of the monitored compounds ranged from 0.83 to 9.57% and the RSD of retention times ranged from 0.0 to 0.7% for all species. In negative mode the peak area RSD ranged from 1.88 to 12.16% and the RSD of retention times ranged from 0.00 to 0.39%. The PCA scores plot of the conditioning injections appear to converge after ca 4–5 injections in positive mode, and after 2 injections in negative mode (see Figs. S1 and S2 in the Supplementary Material). Two of the seven compounds had RSD of peak areas > 10% in the negative conditioning (10.51% and 12.16% for cytidine and tryptophan respectively) but showed a uniform residual distribution around the mean and it was concluded that the instrument was conditioned.

#### Inter- and post-analytical quality control

**Fig. 1:**
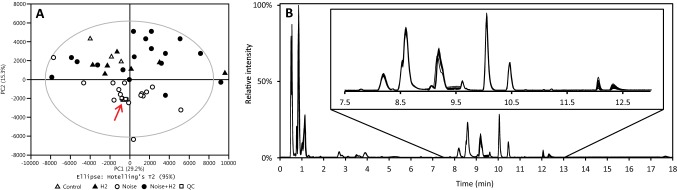
Panel **a**: PCA scores plot of all study samples showing a clear clustering of the 15 repeated QC injections in the center of the first component indicated by the arrow. Panel **b**: commonly scaled and overlaid BPI traces of the 15 repeat QC injections

During the analytical runs, peak areas and retention times of the species used to assess conditioning were monitored successively in the QC sample injections. No sudden irregular shift in peak areas or retention times was observed in either positive or negative mode. Across all QC sample injections, RSD of peak areas and retention times was 3.4–12.3% and 0.04–0.34% respectively in positive mode and 3.4–13.3% and 0.02–0.08% respectively in negative mode. In Fig. 1a the first two components of the PCA scores plot of all study and QC sample injections is presented. The injections of the QC sample all cluster together tightly indicating that the technical variation in the dataset is low in contrast to the biological variation. The QC sample is a composite of all samples in the study which includes a number of sample groups not presented in this paper, this likely explains the slight offset of the cluster from the origin in the plot. Another set of samples included in the QC sample emanated from animals exposed to cisplatin instead of noise and the results of the analysis of these samples is presented elsewhere (Fransson et al. 2017). The PCA scores plot further indicates that there are no potentially outlying samples. In Fig. 1b the base peak ion (BPI) traces of all 15 QC sample injections are presented overlaid with common scaling and exhibit minimal variation in retention times and intensities. The low variation in the monitored species in combination with a low overall variation in the unsupervised analysis show that the protocol is robust with good repeatability and minimal drift in chromatographic performance indicating that the protocol is fit-for-purpose.

### Multivariate data analysis and feature selection

In total, 689 features matched into 276 pcgroups (ESI+) and 1023 features matched into 360 pcgroups (ESI−) were retained with a QC RSD < 30% and retention time between 60 and 800 s.

**Fig. 2:**
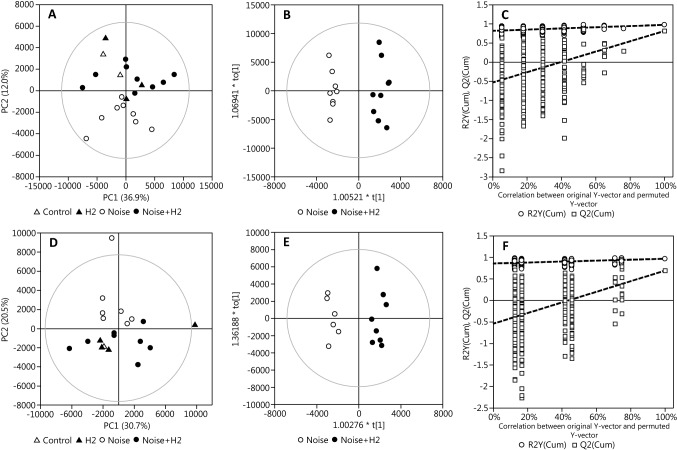
Result of multivariate data analysis of samples from the noise-exposed, left ear and the contralateral, right ear. Panels **a**–**c** and **d**–**f** show the PCA scores plot, OPLS-DA scores plot, and permutation plot of the left and right ear, respectively. There is a clear separation of the Noise group from other groups in the metabolome of both ears. OPLS-DA models on Noise versus Noise + H2 exhibit excellent performance with a clear separation of the groups with no overlap (R2(cum) 0.968, Q2(cum) 0.809 and R2(cum) 0.963, Q2(cum) 0.686 in the left and right ear, respectively). Permutation tests (panels **c** and **f** shows left and right ear, respectively) showed intercepts R2 = 0.815, Q2 = − 0.543 in the left ear and R2 = 0.855, Q2 = − 0.548 in the right ear

Although the impulse noise were administered locally into the left ear canal, perilymph samples were taken from *scala tympani* of both ears and analyzed separately. It is clear from the PCA scores plots of the ESI(+)-data in Figs. 2a, d that there is a systematic difference in the perilymph metabolome of noise-exposed animals not treated (*Noise*, empty circles) and treated with H_2_ (*Noise *+ *H2*, filled circles) in the noise-exposed, left ear. A corresponding effect could also be observed in the contralateral, right ear. Considering the extreme intensity of the impulse noise, a significant effect can be expected also in the right ear, however bone-conducted transmission of sound to the contralateral ear in guinea pigs is poorly investigated. Additionally, the result of the PCA indicate that the H_2_-treated noise-exposed animals are more similar in their metabolic profiles to the *Control* and *H2* groups than is the *Noise* group.

To investigate the effect of H_2_ on noise-exposed animals, OPLS-DA models of *Noise* versus *Noise *+ *H2* were fitted separately on samples from the left or right ears. In Fig. 2b, e are presented the results of models fitted using ESI(+)-data. Both models show a clear separation of the groups (R2 = 0.968, Q2 = 0.809 and R2 = 0.963, Q2 = 0.686 in the left and right ear respectively). The result of permutation tests (n = 500) is presented in Fig. 2c, f with intercepts R2Y = 0.815 and Q2 = − 0.543 in the left ear and R2Y = 0.855 and Q2 = − 0.548 in the right ear showing that the models are reliable. CV-ANOVA tests of the two models yielded p-values p < 0.01 and p = 0.057 for the exposed- and non-exposed ear models respectively. The estimation of classification error by the LOOCV validation described above yielded a CCR of 94% and 87% for the left- and right ear models, respectively (see Supporting Information). No clear group discrimination was obtained using ESI(-)-data.

**Fig. 3:**
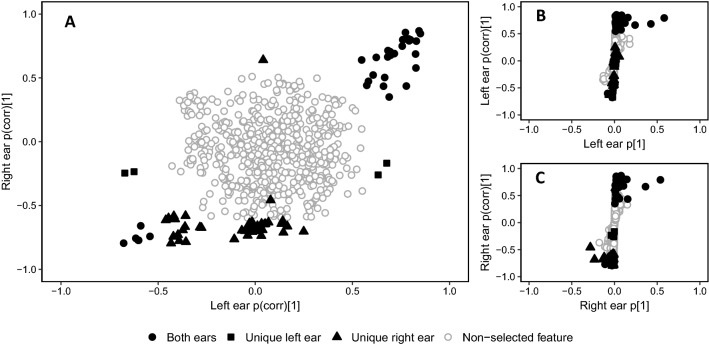
Visualizations of feature predictive loadings as prepared by the SIMCA software. a SUS-plot of the two OPLS-DA models fitted using data from the left ear samples (x-axis) and right ear samples (y-axis). The plot shows the p(corr) value of the respective models as a scatter plot, facilitating the visualization of shared and unique structures in the data. **b**, **c** shows the S-plot of the left and right sample models, respectively. These plots allow the selection of features with a high influence on the model (p[1], x-axis) and high correlation to group classification (p(corr)[1], y-axis). (Wiklund et al. 2008)

From the OPLS-DA models prepared using ESI(+)-data, a total of 85 features were selected (Fig. 3). Following manual verification as described above, 64 features were retained of which 27 were common to samples from both ears and 37 were unique to the right ear samples.

### Metabolite identification and univariate analysis

The selected features were found to correspond to 15 individual chromatographic peaks (Table 1). Stachydrine, homostachydrine, pantothenic acid, creatine and a number of short-chain acyl carnitines could be putatively identified using the online databases HMDb (Wishart et al. 2007, 2009, 2013) and METLIN (Smith et al. 2005). Of these, the identities of stachydrine, creatine, acetylcarnitine, and butyrylcarnitine were confirmed using analytical standards based on retention time and MS/MS spectrum (see Table S3 and Figs. S3 to S19 in Supplementary Information).

**Table 1:** The 15 metabolites selected as important to class discrimination by the multivariate models

Metabolite	MSI identification level^a^	Retention time (s)	Prominent ions (m/z)^b^	Main ion putative molecular formula^c^	Pubchem CID
U137	4	137	156.845, 158.843^e^, 394.606, 396.604, 398.602, 514.487, 516.484, 736.274	n/d^d^	
U169	4	169	253.101	[C_12_H_17_N_2_O_2_S]+	
Pantothenic acid	2	173	90.056, 220.118^e^, 296.0302	[C_9_H_18_NO_5_]+	6613
Butyrylcarnitine	1	450	232.154	[C_11_H_22_NO_4_]+	213144
Homostachydrine	2	493	98.0966, 158.118^e^, 160.123, 180.1, 196.073	[C_8_H_16_NO_2_]+	441447
Stachydrine	1	513	84.081, 102.055, 144.103^e^, 166.084, 182.058	[C_7_H_14_NO_2_]+	115244
U515	4	515	283.164	[C_12_H_24_N_2_O_4_Na]+	
U539	4	539	101.060, 160.134^e^	[C_8_H_18_NO_2_]+	
U547	4	547	116.047, 138.055^e^, 160.037	[C_7_H_8_NO_2_]+	
Acetylcarnitine	1	554	204.124	[C_9_H_18_NO_4_]+	7045767
U569	4	569	262.165	[C_12_H_24_NO_5_]+	
U576	4	576	160.097, 182.080^e^	[C_7_H_13_NO_3_Na]+	
U582 *acylcarnitine	3	582	211.058, 248.150^e^	[C_11_H_22_NO_5_]+	
Creatine	1	628	90.0554, 132.077^e^	[C_4_H_10_N_3_O_2_]+	586
U633 *acylcarnitine	3	633	262.128	[C_11_H_20_NO_6_]+	

Qualitative data shown is the identification level as specified by the MSI, retention time in seconds, prominent mass peaks in the spectrum of the peak, and a putative molecular formula

^a^Metabolite identification level as described by the metabolomics standardization initiative (MSI, Sumner et al. 2007)

^b^Ions determined to be part of spectrum

^c^Determined using the iFit algorithm of the elemental composition tool in MassLynx (ver. 4.1, Waters) and selection of the formula with the highest iFit value

^d^Could not be determined due to the complexity of the spectrum

^e^Base peak

*Putatively determined compound class

**Fig. 4:**
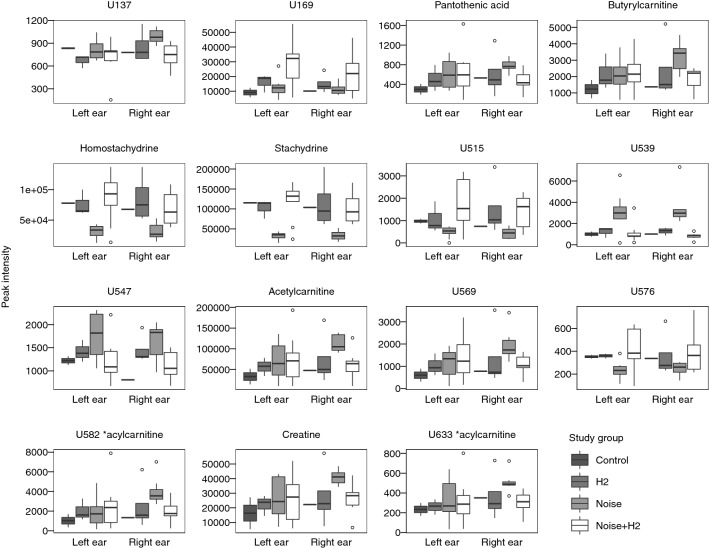
Boxplots of main ion peak intensities of the selected metabolites in the Noise (exposed ear: n = 8, unexposed ear: n = 7), Noise + H2 (exposed ear: n = 9, unexposed ear: n = 8), Control (exposed ear: n = 2, unexposed ear: n = 1), and H2 (exposed ear: n = 3, unexposed ear: n = 4) sample groups. Outlying samples are indicated by open circles

The most intense mass peak of each metabolite was subjected to further univariate consideration and fold changes, VIPpred value, as well as the p value from the univariate significance tests are presented in Table 2. In Fig. 4, boxplots of peak intensities of the most intense mass peak from each metabolite are presented. In short, when contrasting the *Noise* and *Noise *+ *H2* groups, seven metabolites exhibit a different relative intensity in the samples from both ears whereas the other eight only show a corresponding effect in the samples from the right ears only. Stachydrine, homostachydrine, U169, U515, and U576 all exhibit a lower relative intensity in the *Noise* group in contrast to the *Noise *+ *H2* group in both ears, whereas a higher relative intensity is observed for U539, and U547 in both ears. Incidentally, the relative intensity of U169 in the *H2* and *Noise *+ *H2* group is higher than in both the *Control* and *Noise* groups indicating that this is a potential effect of the H_2_ treatment. However, the small number of control group samples (*Control* and *H2*) makes this conclusion tentative.

**Table 2:** Results of the univariate analysis of the main ions of each metabolite spectrum

Metabolite	QC RSD^a^ (%)	m/z^b^	VIPpred^c^		Fold change^d^		p-value^e^	
			Left ear	Right ear	Left ear	Right ear	Left ear	Right ear
U137	6.1	157.845	0.33	0.81	1.15	1.35	4.8E−01	3.7E−03
U169	1.8	253.101	6.27	4.04	0.44	0.51	2.1E−02	1.5E−01
Pantothenic acid	5.7	220.118	0.06	0.92	0.98	1.67	8.2E−01	5.9E−03
Butyrylcarnitine	2.8	232.154	0.14	1.63	0.97	1.70	9.6E−01	4.0E−02
Homostachydrine	3.1	158.118	11.10	9.48	0.36	0.46	7.9E−03	9.3E−03
Stachydrine	2.8	144.103	15.16	14.04	0.27	0.33	2.5E−03	3.1E−04
U515	16.8	283.164	1.64	1.54	0.28	0.31	2.7E−02	1.4E−02
U539	2.8	160.134	2.07	2.88	2.83	4.29	3.6E−02	3.1E−04
U547	7.6	138.055	0.41	0.50	1.43	1.44	4.6E−02	2.9E−02
Acetylcarnitine	3.0	204.124	1.42	7.38	0.94	1.82	1.0E+00	3.7E−03
U569	3.5	262.165	0.32	1.25	0.83	1.80	7.4E−01	5.9E−03
U576	9.1	182.080	0.66	0.51	0.55	0.63	1.5E−02	1.5E−01
U582 *acylcarnitine	3.7	248.150	0.60	1.97	0.77	2.05	6.1E−01	5.9E−03
Creatine	2.6	132.077	0.27	6.10	1.02	1.56	8.9E−01	3.7E−03
U633 *acylcarnitine	6.5	262.128	0.02	0.71	1.05	1.65	8.9E−01	1.2E−03

Presented is the RSD of peak intensities across the 15 QC sample injections, m/z of the peak used for the analysis, VIPpred values determined from the OPLS-DA models, fold changes between groups Noise and Noise + H2, and p-value determined from a Wilcoxon rank-sum test of Noise versus Noise + H2

^a^Peak area RSD over QC sample injections

^b^m/z of ion used for univariate analysis

^c^Variable of importance in the predictive component of the OPLS-DA model

^d^Fold change calculated as mean peak area in Noise sample group divided by mean peak area in Noise + H2 sample group

^e^Two-sided wilcoxon rank-sum test of Noise versus Noise + H2

*Putatively determined compound class

An additional eight metabolites were found to have a differentiating relative intensity in only the right ear samples. Pantothenic acid, creatine, butyrylcarnitine, acetylcarnitine, two unidentified acylcarnitines, U137, and U569 all had a higher relative intensity in the *Noise* group in contrast to the *Noise *+ *H2* group.

### Biological interpretation

#### Noise-induced hearing loss

Elevated levels of ROS follow the formation of excess amounts of superoxide inside the mitochondria of the hair cells via a few identified pathways (Henderson et al. 2006): (i) overdriving of the mitochondria due to noise stimulation, (ii) excitotoxic activity by excessive neurotransmitter release into the hair cell synapses initiating apoptotic cell death further straining aerobic respiration, and (iii) a reduction in cochlear blood flow following noise exposure leading to hypoxia that lowers reaction efficiencies inside the mitochondria further exacerbating superoxide formation. Animals receiving H_2_ treatment (*Noise *+ *H2* group*)* after impulse noise exposure exhibited less hearing loss than animals in the *Noise* group (Videhult Pierre et al. Hydrogen gas inhalation attenuates acute noise trauma. A preclinical in vivo study, *in manuscript*). Thus, inhalation of H_2_ immediately after impulse noise trauma had a rescue effect on the cochlea and most probably on the cytotoxic mechanisms induced by ROS. As we have shown, this protective effect can also be seen in the perilymph metabolome. As the animals in the present study were allowed to survive only 4 days after impulse noise exposure the nature of hearing loss ought to be defined as acute auditory dysfunction.

#### Changes in acyl carnitines

The main role of acyl carnitines inside the cell is to facilitate transport of long-chain fatty acids across the mitochondrial membrane for subsequent beta-oxidation and energy production (Bieber and Choi 1977; Bender 2012; Longo et al. 2016; Traina 2016). In addition, acyl carnitines may also be formed in the catabolism of amino acids. As an example, the amino acids valine, isoleucine, and leucine all undergo catabolism that leads to acyl-CoA substrates that may form acyl carnitines (Bieber and Choi 1977; Violante et al. 2013). As has been shown, the levels of various acyl carnitines in the perilymph metabolome of the *Noise* group was higher than the levels observed in samples from the *Noise *+ *H2*, *Control*, and *H2* groups. The increased levels of acyl carnitines are indicative of an increase in oxidative stress, which indicates that the metabolic strain caused by the noise exposure is attenuated by the administration of H_2_.

#### Changes in stachydrine and homostachydrine

Stachydrine and homostachydrine belong to a group of compounds known as osmoprotectants that help regulate cell volume (Lang 2007). Stachydrine is known to act as an osmoprotectant for *E. coli* and other bacteria in human urine and has also been detected in human blood (Chambers and Kunin 1987). However, to the best of our knowledge, no pathway for the formation of stachydrine or homostachydrine in mammals is known. They are a common component of many plants and their occurrence in mammals is likely explained by intake of such plants as food (Lever et al. 1994). The lower relative intensity of stachydrine and homostachydrine in the *Noise* group in contrast with the *Noise *+ *H2*, *Control*, and *H2* groups indicate the presence of osmolytic responses in the absence of H_2_ treatment. The *Noise *+ *H2* group is again similar to the *Control* and *H2* groups, which show that the H_2_ attenuate the effects of the noise exposure. Incidentally, stachydrine and other osmoprotectants have not been previously shown to have a roll in hearing loss.

## Conclusions

An LCMS-based method has been presented for untargeted metabolomics of perilymph applied to samples taken from a guinea pig model of H_2_ attenuation of NIHL. Furthermore, it has been shown that the protocol is fit-for-purpose as the combination of targeted and untargeted quality control methods indicate a low analytical variation, as indicated by the low RSD in both peak areas and retention times as well as the tight clustering of the QC samples in the unsupervised data analysis. Supervised analysis of the obtained data afforded robust and highly discriminatory models indicating a number of metabolites as important including two osmoprotectants, a number of short-chain acyl carnitines and a number of other metabolites. Changes in the perilymph metabolome were found in samples taken from both ears indicating a bilateral effect, despite the local administration of noise into the left ear. For the most part, these effects appeared to originate from dysregulation in response to noise exposure as the H_2_-treated group appeared more similar in the models to the animals not exposed to noise. Thus, the results indicated that the increased strain on aerobic respiration caused by the noise exposure was attenuated by H_2_.

## Electronic supplementary material

Below is the link to the electronic supplementary material.

Supplementary material 1 (DOCX 684 kb)

## Abbreviations

ABR: Auditory brainstem response
ACN: Acetonitrile
BPI: Base peak ion
CCR: Correct classification rate
CSF: Cerebrospinal fluid
H_2_: Hydrogen gas
ESI: Electrospray ionization
LCMS: Liquid chromatography mass spectrometry
LOOCV: Leave-one-out cross validation
MVDA: Multivariate data analysis
NIHL: Noise-induced hearing loss
OPLS-DA: Orthogonal projection to latent structures discriminant analysis
PCA: Principal component analysis
QC: Quality control
QTOF: Quadrupole time of flight
ROS: Reactive oxygen species
S/N: Signal to noise
SUS plot: Shared and unique structures plot
UPLC: Ultra performance liquid chromatography
