# Supplementary material for: An LCMS-based untargeted metabolomics protocol for cochlear perilymph: highlighting metabolic effects of hydrogen gas on the inner ear of noise exposed Guinea pigs
Source: Metabolomics. 2019 Oct 5;15(10):138. doi: 10.1007/s11306-019-1595-1 (PMC6778533; doi:10.1007/s11306-019-1595-1)
Supplement: Supplementary file 1 — Supplementary material 1 (DOCX 684 kb) [file 11306_2019_1595_MOESM1_ESM.docx]

Electronic supplementary material

# An LCMS-based untargeted metabolomics protocol for cochlear perilymph: Highlighting metabolic effects of hydrogen gas administration on the inner ear of noise exposed guinea pigs

Kristian Pirttilä^1,*^, Pernilla Videhult Pierre^2^, Jakob Haglöf^1^, Mikael Engskog^1^, Mikael Hedeland^1^, Göran Laurell^3^, Torbjörn Arvidsson^1^, Curt Pettersson^1^

# Affiliations

^1^ Department of Medicinal Chemistry, Uppsala University, Uppsala, Sweden

^2^ Division of Audiology, Department of Clinical Science, Intervention and Technology, Karolinska Institutet, Stockholm, Sweden

^3^ Department of Surgical Science, Uppsala University, Uppsala, Sweden

^*^ Correspondence: [kristian.pirttila@ilk.uu.se](mailto:kristian.pirttila@ilk.uu.se)

# Sample analysis

## Tune settings

ESI+: capillary voltage 1 kV, source temperature 120 °C, sampling cone voltage 30 V, source offset voltage 50 V, source gas flow 0 mL/min, desolvation gas temperature 500 °C, cone gas flow 50 L/h, desolvation gas flow 800 L/h, nebulized gas pressure 6 bar, ESI-: capillary voltage 2 kV, source temperature 120 °C, sampling cone voltage 25 V, source offset voltage 60 V, source gas flow 0 mL/min, desolvation gas temperature 450 °C, cone gas flow 50 L/h, desolvation gas flow 800 L/h, nebulized gas pressure 6 bar.

## Nonlinear gradient

The nonlinear gradient used (setting 8 in MassLynx) was calculated as a cubic function according to %B(t) = %B_i_ + [(%B_f_ - %B_i_) * X^3^], where %B_i_ is the initial setting, %B_f_ is the final setting, and X = (t - T_i_) / (T_f_ - T_i_), where T_i_ is the initial time, and T_f_ is the final time.

## Substances used to monitor conditioning and instrument stability

In positive mode; Choline (m/z 104.1072), Nicotinic acid (m/z 122.0248), Cytidine (m/z 266.0747), Glycine betaine (m/z 118.0863), Phenylalanine (m/z 120.0813), Tryptophan (m/z 188.0713), Taurine (m/z 169.9858), and Glutamine (m/z 130.0496) was used.

In negative mode; Uridine (m/z 243.0623), Succinic acid (m/z 117.0193), Cytidine (m/z 242.0782), Phenylalanine (m/z 164.0717), Tryptophan (m/z 203.0826), Taurine (m/z 124.0074), and Glutamine (m/z 145.0619) was used.

# Data preprocessing

## IPO starting parameters

Starting parameters used with the IPO package for optimization of XCMS was min_peakwidth 10-20, max_peakwidth 26-42 and keeping ppm value constant at 20 ppm.

## XCMS parameters

Table S1 XCMS parameters used for peak detection, grouping, peak alignment, and peak filling.

| **Function** | **Parameter** | **ESI+** | **ESI-** |
| --- | --- | --- | --- |
| xcmsSet | peakwidth | 5-50 | 7-24 |
|  | ppm | 20 | 20 |
|  | snthresh | 0 | 0 |
|  | mzdiff | 0.02 | 0.05 |
|  | prefilter | {3, 100} | {3, 100} |
|  | mzCenterFun | “wMean” | “wMean” |
| group | bw | 12.4 | 1 |
|  | mzwid | 0.02 | 0.02 |
|  | minfrac | 1 | 0.6 |
|  | minsamp | 1 | 1 |
|  | max | 50 | 50 |
| retcor | method | “obiwarp” | “obiwarp” |
|  | distFun | “cor_opt” | “cor_opt” |
|  | response | 1 | 1 |
|  | gapInit | 0.4 | 0.38 |
|  | gapExtend | 2.7 | 2.4 |
|  | localAlignment | 0 | 0 |
| fillPeaks | *Default parameters were used* | | |

## CAMERA parameters

Table S2 CAMERA parameters used for adduct and fragment annotation.

| **Function** | **Parameter** | **ESI+** | **ESI-** |
| --- | --- | --- | --- |
| groupFWHM | perfwhm | 0.6 | 0.6 |
| findIsotopes | mzabs | 0.01 | 0.01 |
|  | intval | “into” | “into” |
|  | minfrac | 0.2 | 0.2 |
| groupCorr | cor_eic_th | 0.8 | 0.8 |
|  | cor_exp_th | 0.9 | 0.9 |
|  | calcIso | TRUE | TRUE |
|  | calcCiS | TRUE | TRUE |
| findAdducts | ppm | 5 | 5 |
|  | mzabs | 0.01 | 0.01 |

# Quality control

## Pre-analytical quality control

### System suitability testing

### Instrument conditioning

Figure S1 and S2 show the PCA scores plots of the positive and negative conditioning injections prepared as part of the quality control scheme. System conditioning was considered adequate when a clear convergence of the scores in both PC1 and PC2 was observed. It is clear that the scores converge after ca 5-6 injections (Figure S1) in positive mode and after 2 injections in the negative mode (Figure S2). This is expected as the system already was partially conditioned for the negative mode analysis.

1

2

3

4

5

6

7

8

9

10

11

12

P

C

2

(

2

6

.

0

%

)

-40

-20

0

20

40

-60

-40

-20

0

20

40

60

PC1 (54.5%)

Figure S1 PCA scores plot of the 12 conditioning injections in positive mode showing a convergence of the scores after ca 5-6 injections.

1

2

3

4

5

6

7

8

9

10

11

12

P

C

2

(

1

2

.

0

%

)

-15

-10

-5

0

5

10

15

-20

-15

-10

-5

0

5

10

15

20

PC1 (42.5%)

Figure S2 PCA scores plot of the 12 conditioning injections in negative mode showing a convergence of the scores after 2 injections.

## OPLS-DA model classification error estimation

### Validation scheme

The estimation of classification error of the OPLS-DA models was performed in the R statistical language as described below.

1. The data set was split according leave-one-out cross validation into n=17 and n=15 folds for the left and right ear samples, respectively.
2. For each training set containing n-1 samples an OPLS-DA model was fitted using 7-fold cross validation to select the model parameters and the held out sample was predicted.
3. The correct classification rate was determined as the ratio of the correctly classified samples over the total number of samples in that sample group.

### Validation results

### Left ear

The LOOCV validation described above for the left ear samples resulted in a correct classification rate of 16/17 = 94% as can be seen in Table 1 below.

Table 1 Confusion matrix showing the classification error for the left ear samples, as determined by the LOOCV validation described above

|  | Noise+H2 | Noise |
| --- | --- | --- |
| Noise+H2 | 8 | 1 |
| Noise | 0 | 8 |

### Right ear

The LOOCV validation described above for the right ear samples resulted in a correct classification rate of 13/15 = 86% as can be seen in Table 2 below.

Table 2 Confusion matrix showing the classification error for the right ear samples, as determined by the LOOCV validation described above

|  | Noise+H2 | Noise |
| --- | --- | --- |
| Noise+H2 | 7 | 1 |
| Noise | 1 | 6 |

# Confirmation of putative identities using analytical standards

## Methods

## Preparation of solvents and samples

#### Preparation of standard mixture

To a 836.0 µl acetonitrile was added 9.0 µl acetylcarnitine (115.8 µM), 27.0 µl butyrylcarnitine (37.4 µM), 110.0 µl creatine (181.6 µM), 18.0 µl ^13^C_2_-Stachydrine (55.9 µM).

#### Preparation of 200 mM ammonium formate + 2.5 vol% formic acid buffer

1266.2 mg LCMS-grade ammonium formate (Thermo Scientific, optima) was weighed in a 100 ml volumetric flask and dissolved in Milli-Q water. 2.5 ml LCMS-grade formic acid (Thermo Scientific, optima) was added and the pH checked to be ~3. The mixture was diluted to the mark with Milli-Q water.

#### Preparation of mobile phase A (5 mM HCOONH4 + 0.0625% FA in 95:5 ACN:MQ)

To a 1000 ml volumetric flask was added 25 ml ammonium formate buffer (200 mM, pH~3) and 25 ml Milli-Q water. Acetronitrile was added to the mark and the mixture was degassed in an ultrasonic bath prior to use.

#### Preparation of mobile phase B (5 mM HCOONH4 + 0.0625% FA in 5:95 ACN:MQ)

To a 1000 ml volumetric flask was added 25 ml ammonium formate buffer (200 mM, pH~3) and 50 ml Acetonitrile. Milli-Q water was added to the mark and the mixture was degassed in an ultrasonic bath prior to use.

#### Preparation of wash solutions

A 95:5 mixture of Acetonitrile:water was used as strong wash solution and prepared by measuring 950 ml Acetonitrile in a 1000 ml measuring cylinder and adding Milli-Q water to the 1000 ml mark. A 10:90 mixture of Acetonitrile:water was used as weak wash solution and prepared by measuring 900 ml Milli-Q water in a 1000 ml measuring cylinder and adding Acetonitrile to the 1000 ml mark.

### LC-ESI-QTOF analysis

## Results

### MS/MS analysis

Table S3 Summary of authentic standard analysis for metabolite identification.

| Metabolite | t_R_, standard (min) | t_R_, QC injection (min) | Collision energy | MS/MS spectrum, standard injection | MS/MS spectrum, QC sample injection |
| --- | --- | --- | --- | --- | --- |
| Acetylcarnitine | 8.87 | 8.86 | 0V | 85.029 (12.0%), 145.051 (8.6%), 204.124 (100.0%), 205.127 (10.3%) | 85.029 (9.6%), 145.050 (7.9%), 204.124 (100.0%), 205.127 (8.2%) |
|  |  |  | 10V | 85.029 (12.0%), 145.051 (8.6%), 204.124 (100.0%), 205.127 (10.3%) | 85.029 (9.6%), 145.050 (7.9%), 204.124 (100.0%), 205.127 (8.2%) |
|  |  |  | 20V | 84.082 (11.0%), 85.030 (100.0%), 144.103 (43.8%), 204.124 (12.6%) | 84.082 (10.5%), 85.030 (100.0%), 144.103 (43.9%), 204.124 (12.6%) |
|  |  |  | 40V | 84.081 (5.4%), 85.029 (100.0%), 144.101 (10.5%) | 51.584 (18.5%), 85.030 (100.0%), 144.095 (17.1%) |
| Butyrylcarnitine | 7.62 | 7.61 | 0V | 85.029 (6.3%), 173.082 (9.0%), 232.157 (100.0%), 233.158 (13.9%) | 85.029 (2.7%), 232.155 (100.0%), 233.158 (3.9%) |
|  |  |  | 10V | 85.029 (25.2%), 173.081 (12.5%), 232.155 (100.0%), 233.158 (11.5%) | 85.029 (25.4%), 173.082 (16.7%), 232.154 (100.0%), 233.152 (11.8%) |
|  |  |  | 20V | 85.029 (100.0%), 144.102 (34.7%), 232.154 (14.8%) | 85.029 (100.0%), 144.102 (44.5%), 232.166 (20.1%) |
|  |  |  | 40V | 84.082 (21.0%), 85.029 (100.0%), 144.102 (9.8%) | N/D |
| Creatine | 9.92 | 9.91 | 0V | 90.055 (27.8%), 132.077 (100.0%) | 90.056 (27.1%), 132.078 (100.0%) |
|  |  |  | 10V | 90.056 (43.9%), 114.068 (4.2%), 132.077 (100.0%) | 90.056 (39.7%), 114.067 (3.0%), 132.078 (100.0%) |
|  |  |  | 20V | N/D | N/D |
|  |  |  | 40V | N/D | N/D |
| Standard: ^13^C_2_-stachydrine  QC: stachydrine | 7.54 | 7.53 | 0V | 146.110 (100.0%), 147.113 (6.3%), 168.092 (12.7%), 184.065 (11.1%) | 144.103 (100.0%), 145.106 (5.5%), 166.085 (9.1%), 182.058 (14.6%) |
|  |  |  | 10V | 146.110 (100.0%), 147.112 (5.4%) | 144.103 (100.0%), 145.106 (7.1%) |
|  |  |  | 20V | 86.089 (4.1%), 146.110 (100.0%), 147.112 (5.6%) | 84.082 (5.4%), 144.102 (100.0%), 145.105 (8.3%) |
|  |  |  | 40V | 86.089 (10.0%), 146.109 (100.0%), 147.110 (6.5%) | 84.080 (10.0%), 144.102 (100.0%), 145.105 (9.5%) |

### Acetylcarnitine


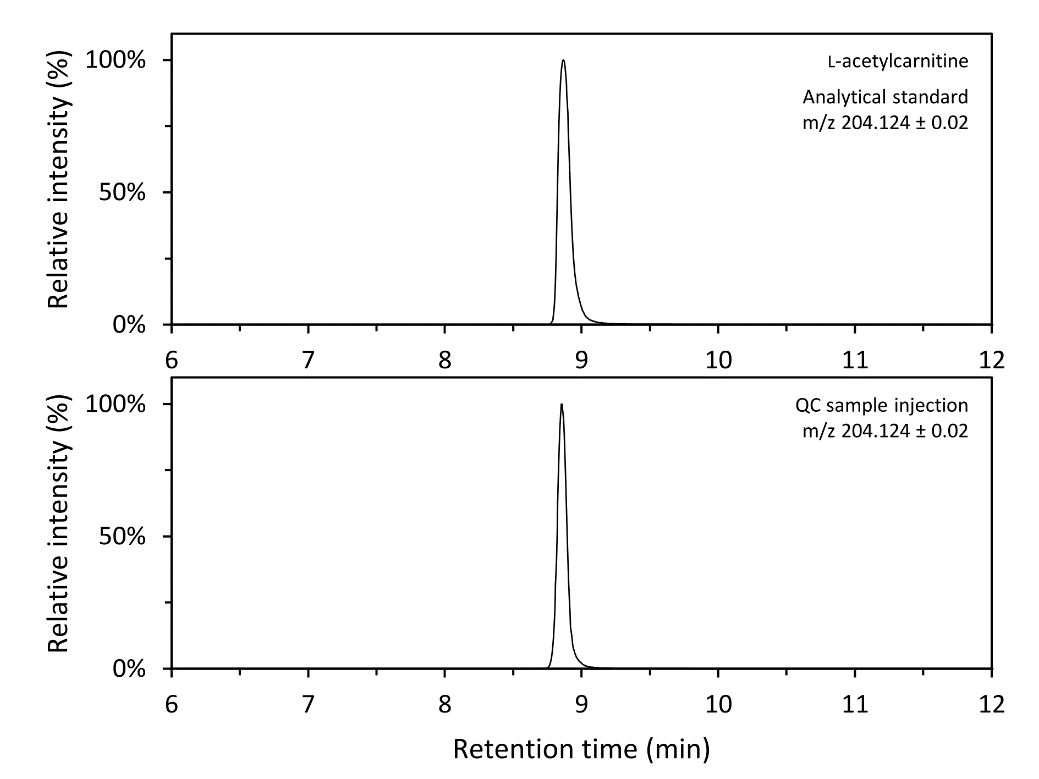


Figure S3 Extracted ion chromatogram of acetylcarnitine (m/z 204.124 ± 0.02). Upper: analysis of analytical standard. Lower: QC sample injection.


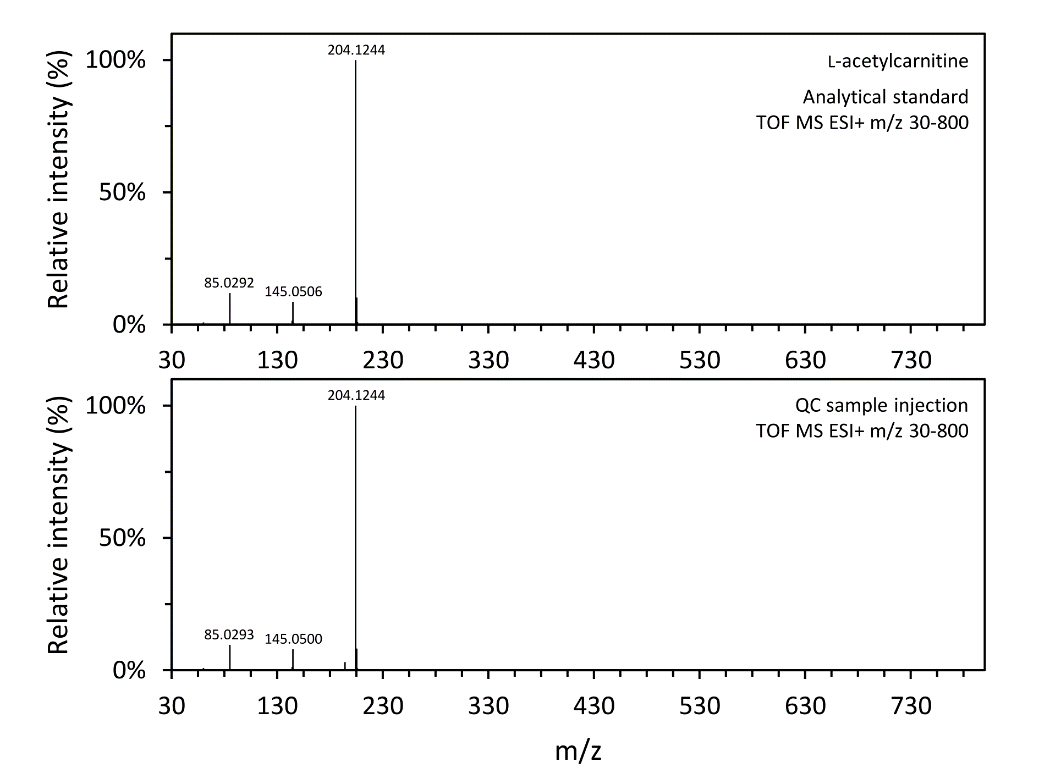


Figure S4 TOF MS ESI+ spectrum of acetylcarnitine. Upper: analysis of analytical standard. Lower: QC sample injection.


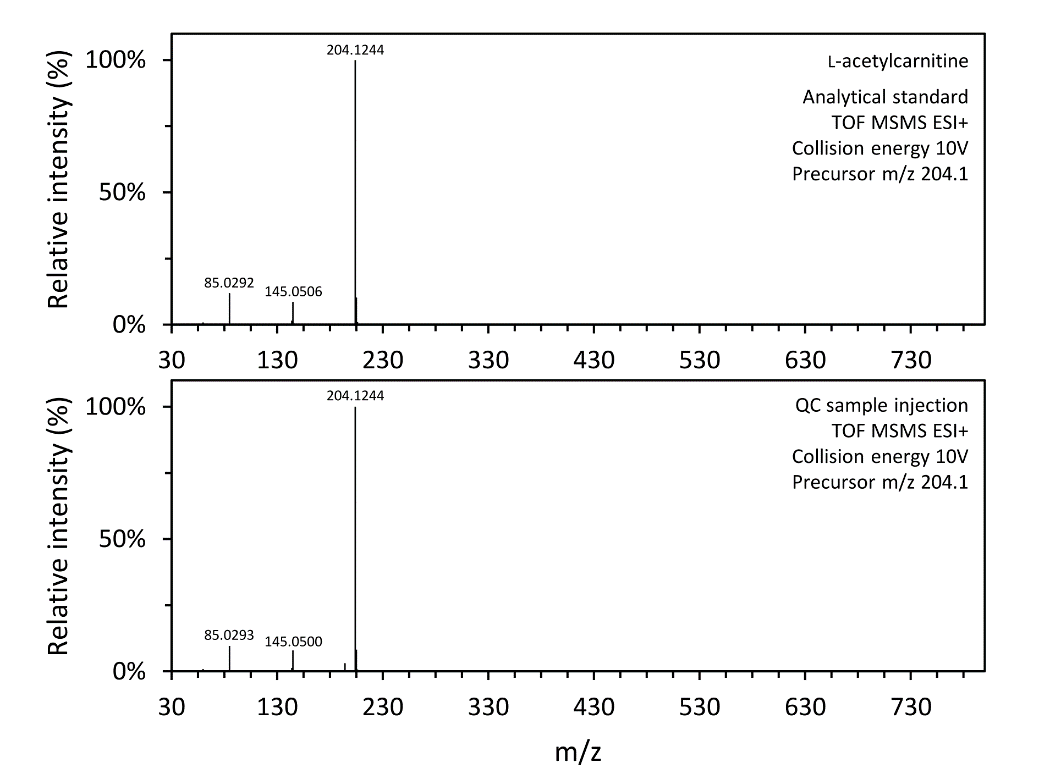


Figure S5 MS/MS spectrum of acetylcarnitine. Precursor ion m/z 204.1, collision energy 10V. Upper: analysis of analytical standard. Lower: QC sample injection.


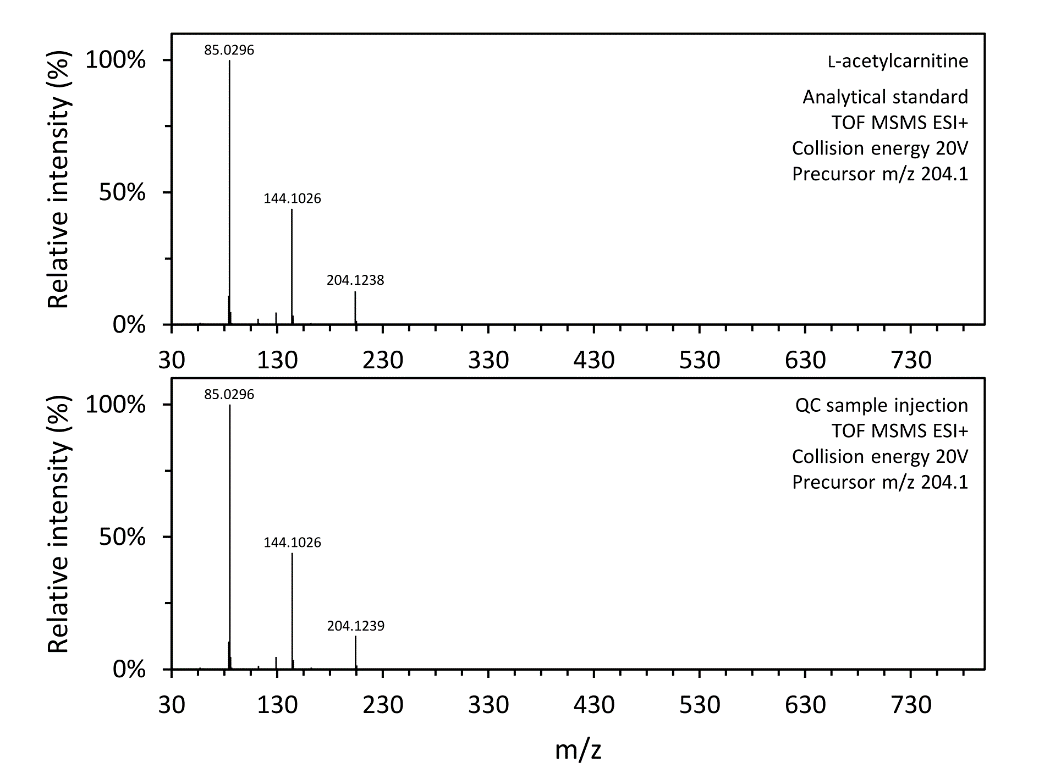


Figure S6 MS/MS spectrum of acetylcarnitine. Precursor ion m/z 204.1, collision energy 20V. Upper: analysis of analytical standard. Lower: QC sample injection.


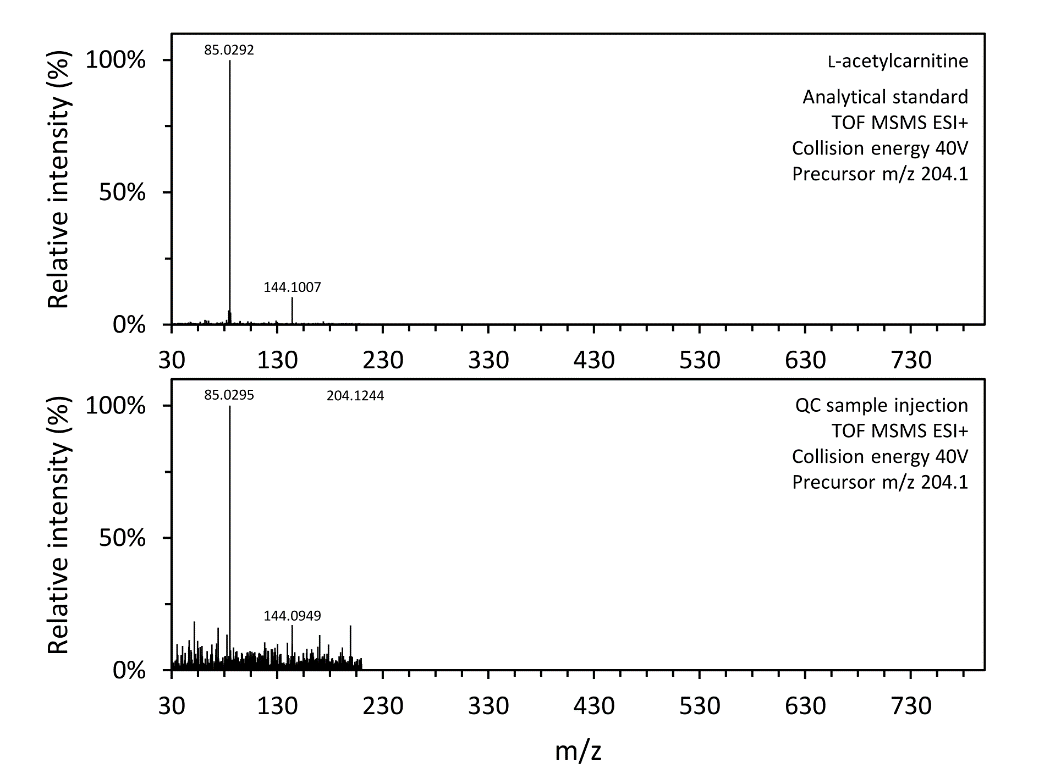


Figure S7 MS/MS spectrum of acetylcarnitine. Precursor ion m/z 204.1, collision energy 40V. Upper: analysis of analytical standard. Lower: QC sample injection.

### Butyrylcarnitine


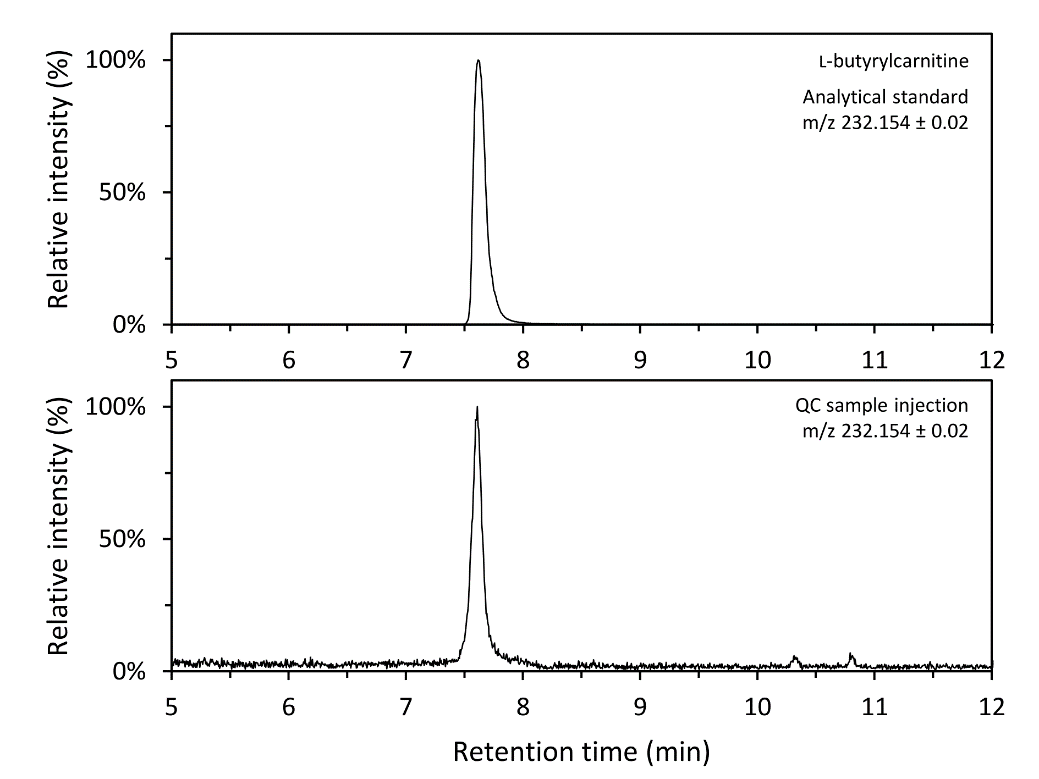


Figure S8 Extracted ion chromatogram of butyrylcarnitine (m/z 232.154 ± 0.02). Upper: analysis of analytical standard. Lower: QC sample injection.


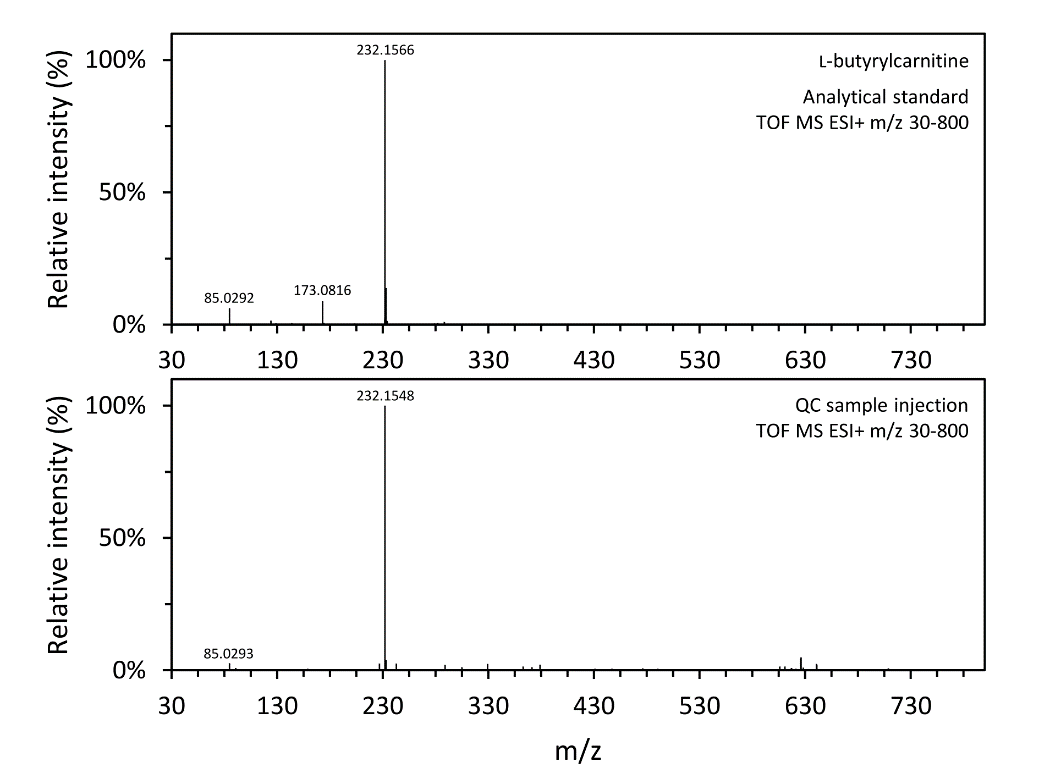


Figure S9 TOF MS ESI+ spectrum of butyrylcarnitine. Upper: analysis of analytical standard. Lower: QC sample injection.


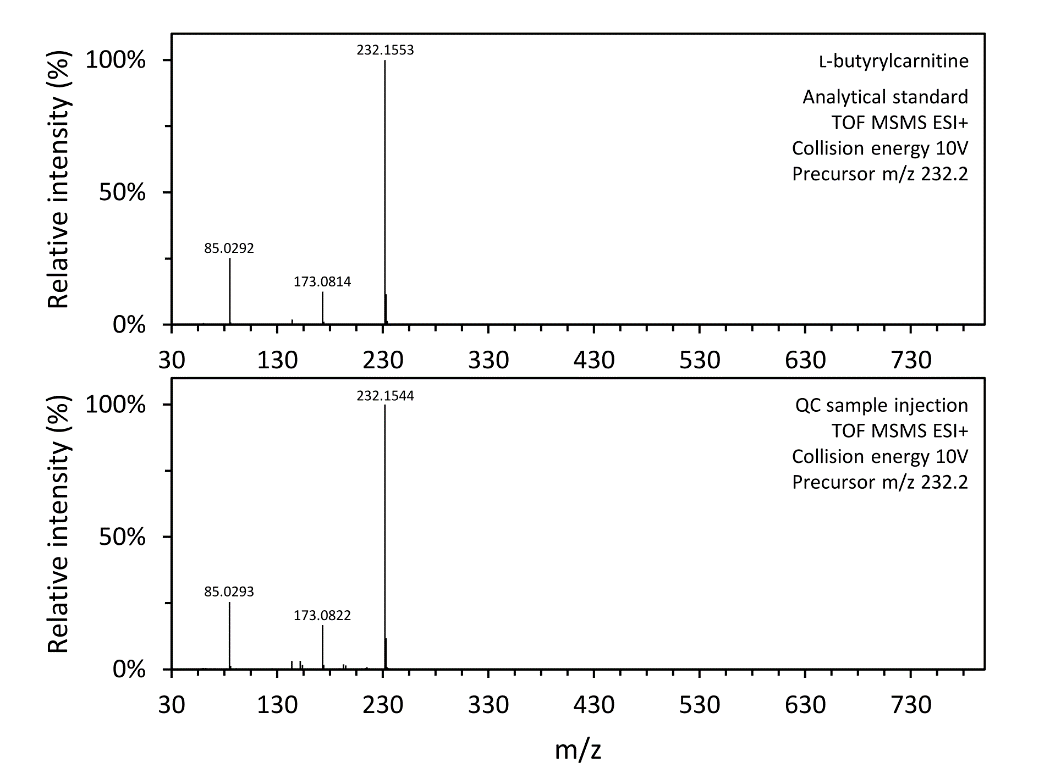


Figure S10 MS/MS spectrum of butyrylcarnitine. Precursor ion m/z 232.2, collision energy 10V. Upper: analysis of analytical standard. Lower: QC sample injection.


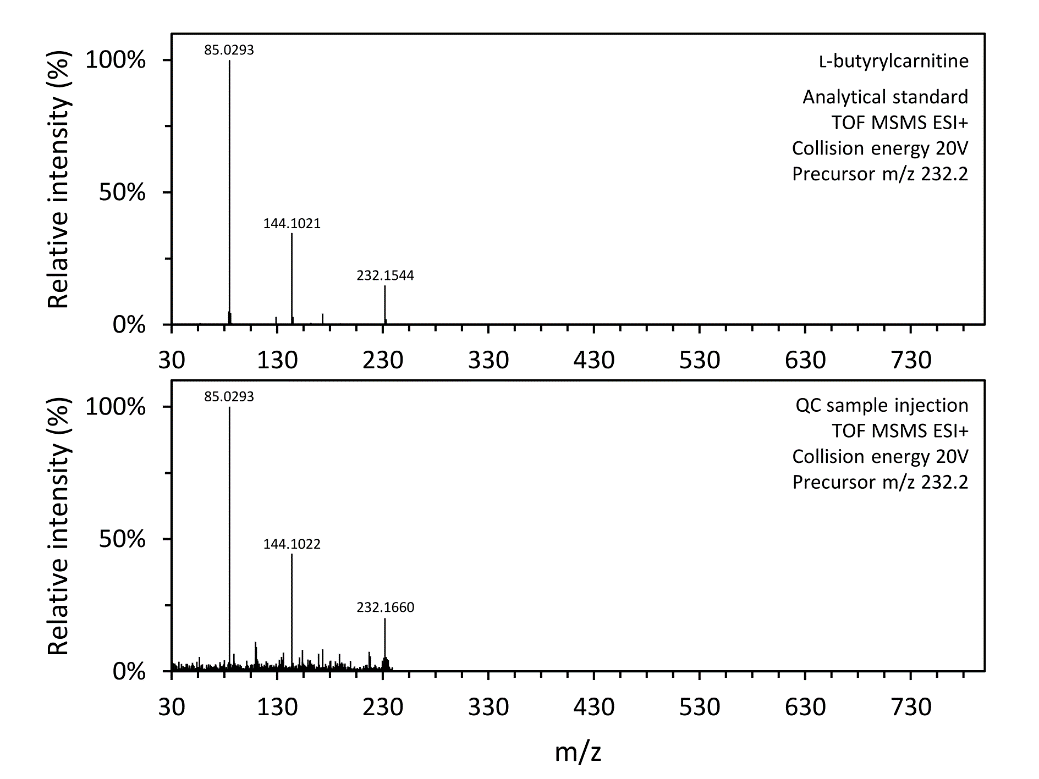


Figure S11 MS/MS spectrum of butyrylcarnitine. Precursor ion m/z 232.2, collision energy 20V. Upper: analysis of analytical standard. Lower: QC sample injection.

No MS/MS spectrum was acquired for butyrylcarnitine at collision energy 40V due to poor signal.

### Creatine


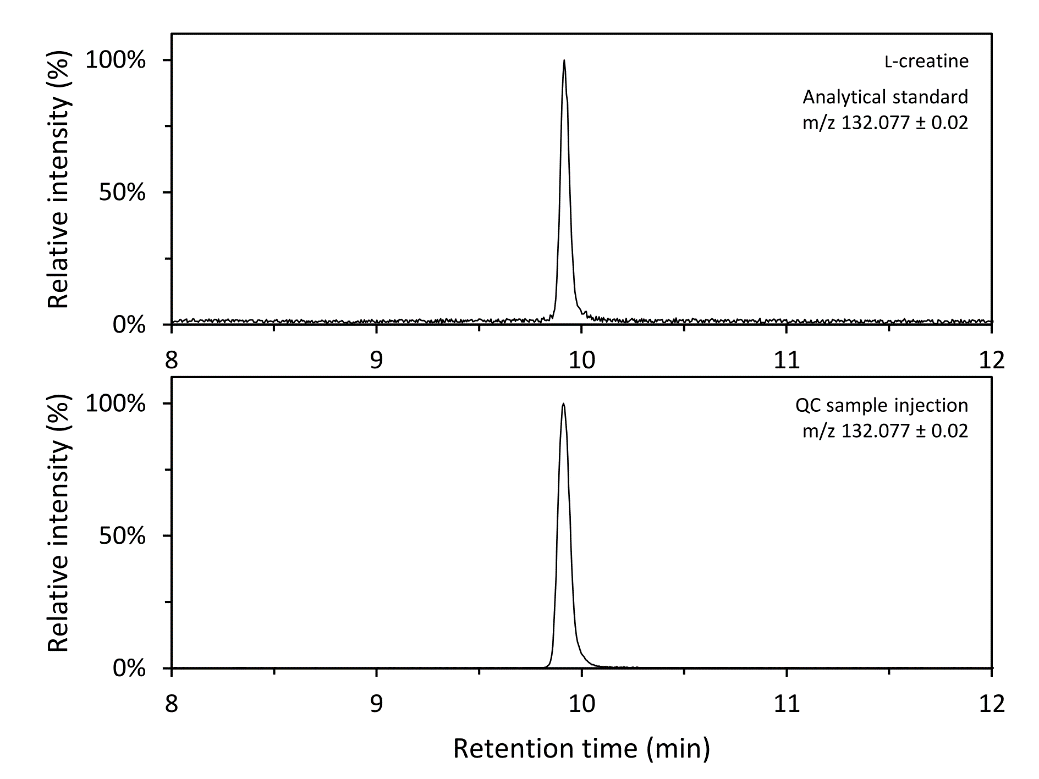


Figure S12 Extracted ion chromatogram of creatine (m/z 132.077 ± 0.02). Upper: analysis of analytical standard. Lower: QC sample injection.


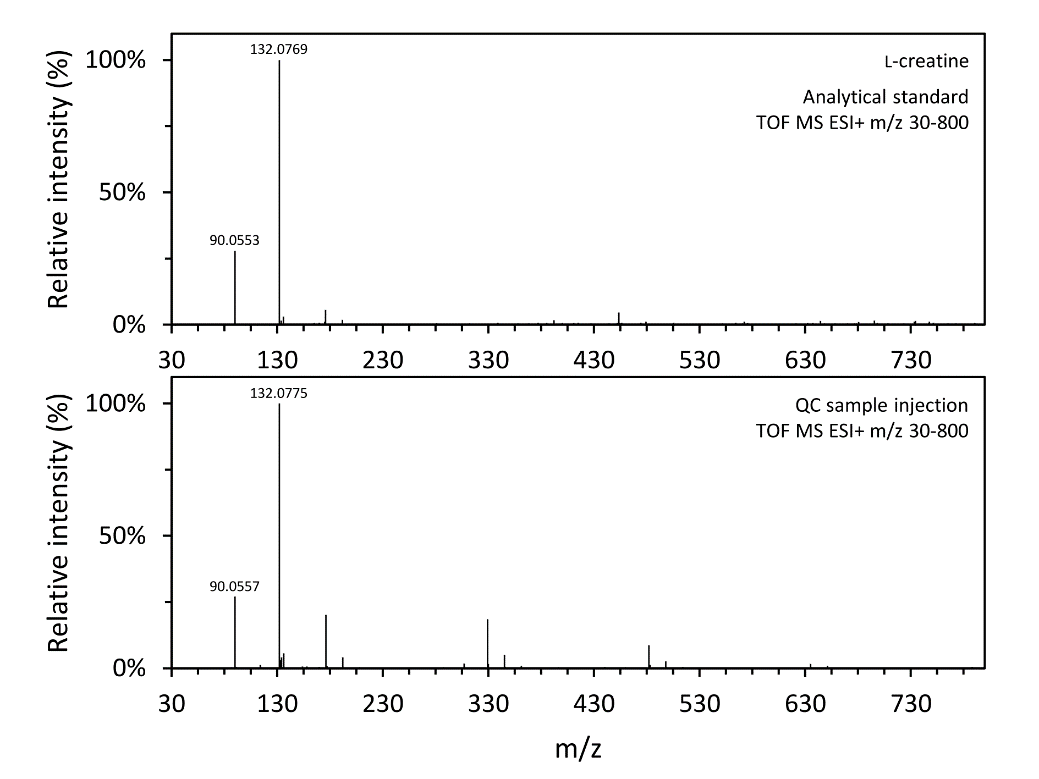


Figure S13 TOF MS ESI+ spectrum of creatine. Upper: analysis of analytical standard. Lower: QC sample injection. The additional ions in the QC sample injection at m/z 176.0412, 329.0931, and 482.1451 correspond to [M+2Na-H]^+^, [2M+3Na-2H]^+^, [3M+4Na-3H]^+^ respectively.
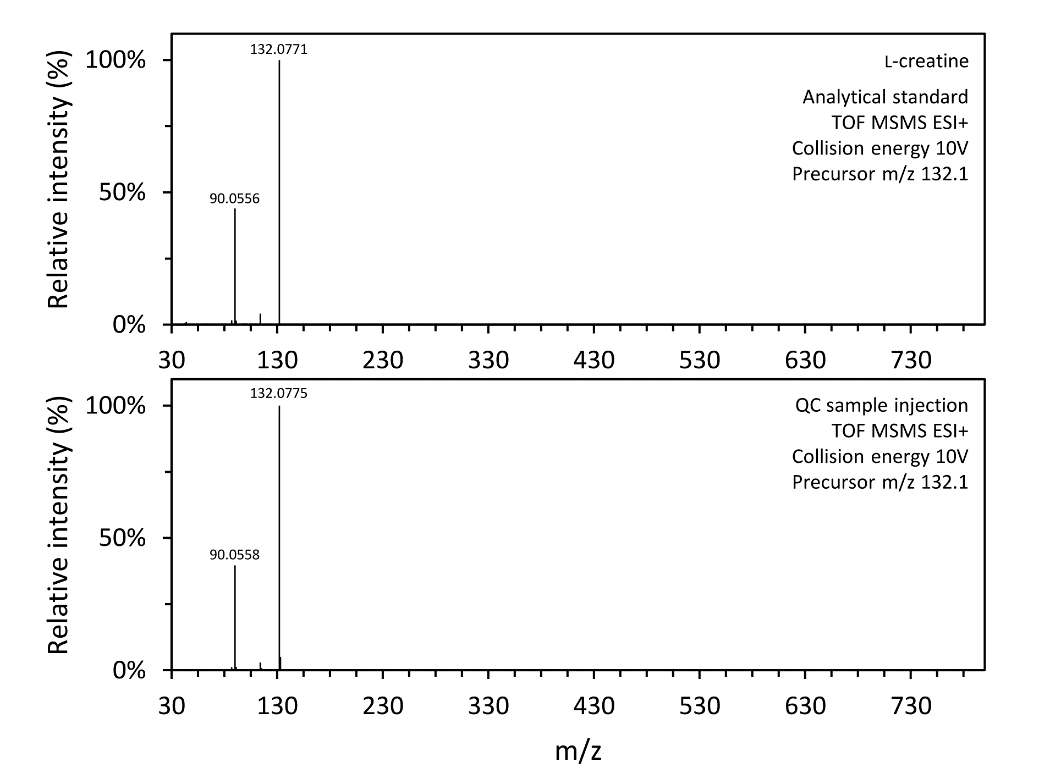


Figure S14 MS/MS spectrum of butyrylcarnitine. Precursor ion m/z 132.1, collision energy 10V. Upper: analysis of analytical standard. Lower: QC sample injection.

No MS/MS spectrum was acquired at collision energies 20V and 40V due to poor signal.

### Stachydrine


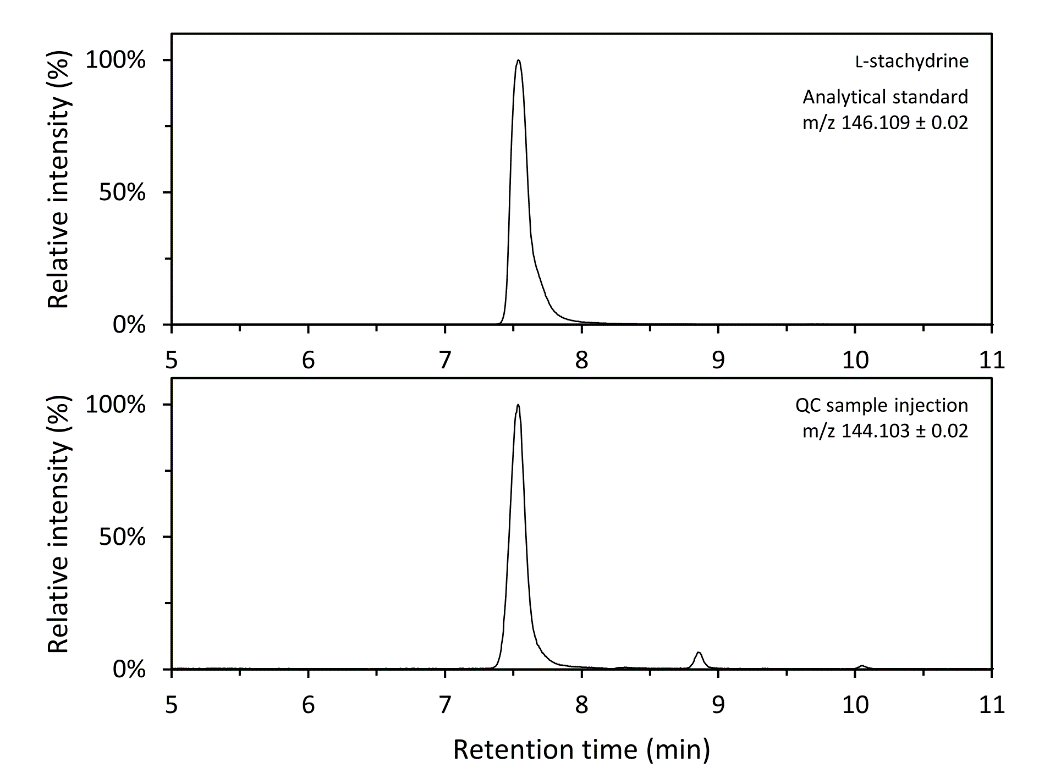


Figure S15 Extracted ion chromatogram of stachydrine. Upper: analysis of analytical standard isotope labelled (13C2)-stachydrine (m/z 146.109 ± 0.02). Lower: QC sample injection (m/z 144.103 ± 0.02).


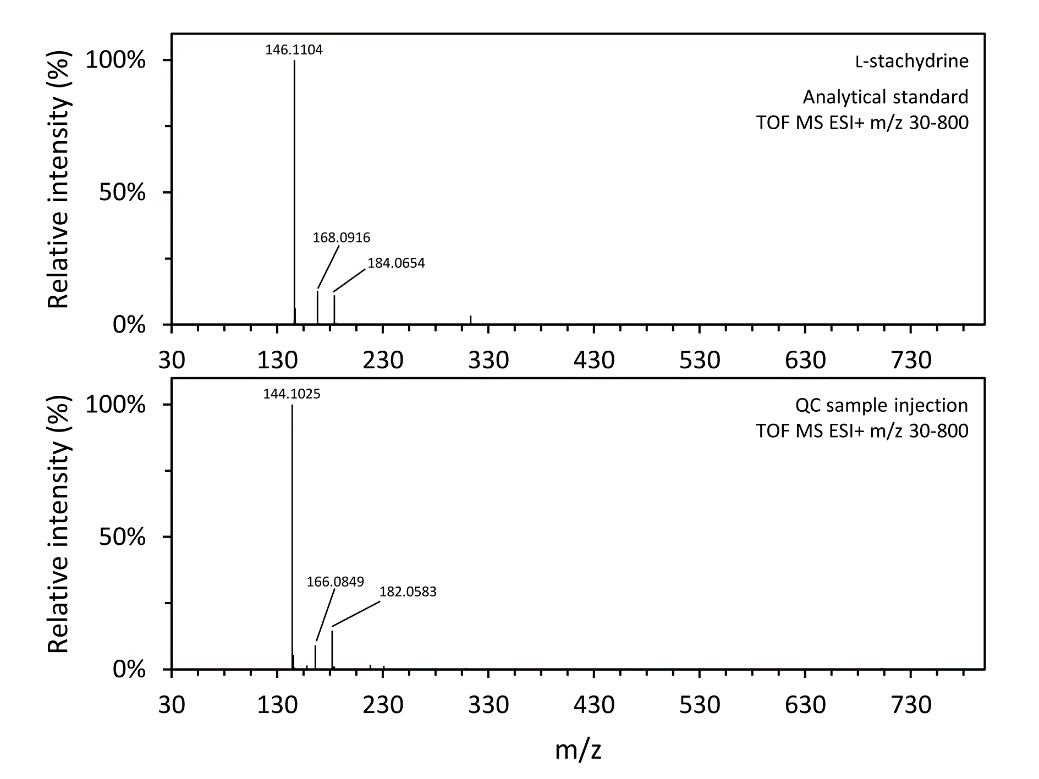


Figure S16 TOF MS ESI+ spectrum of stachydrine. Upper: analysis of analytical standard (13C2)-stachydrine. Lower: QC sample injection.


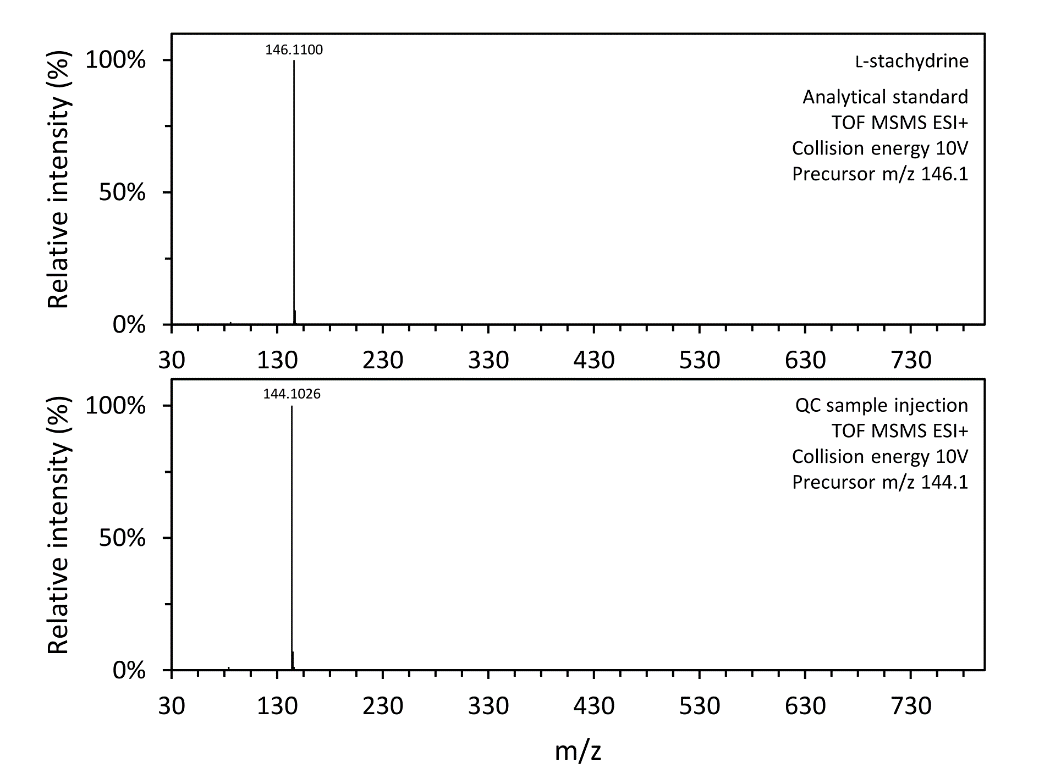


Figure S17 MS/MS spectrum of stachydrine. Collision energy 10V. Upper: analysis of analytical standard (13C2)-stachydrine. Precursor ion m/z 146.1. Lower: QC sample injection. Precursor ion 144.1.


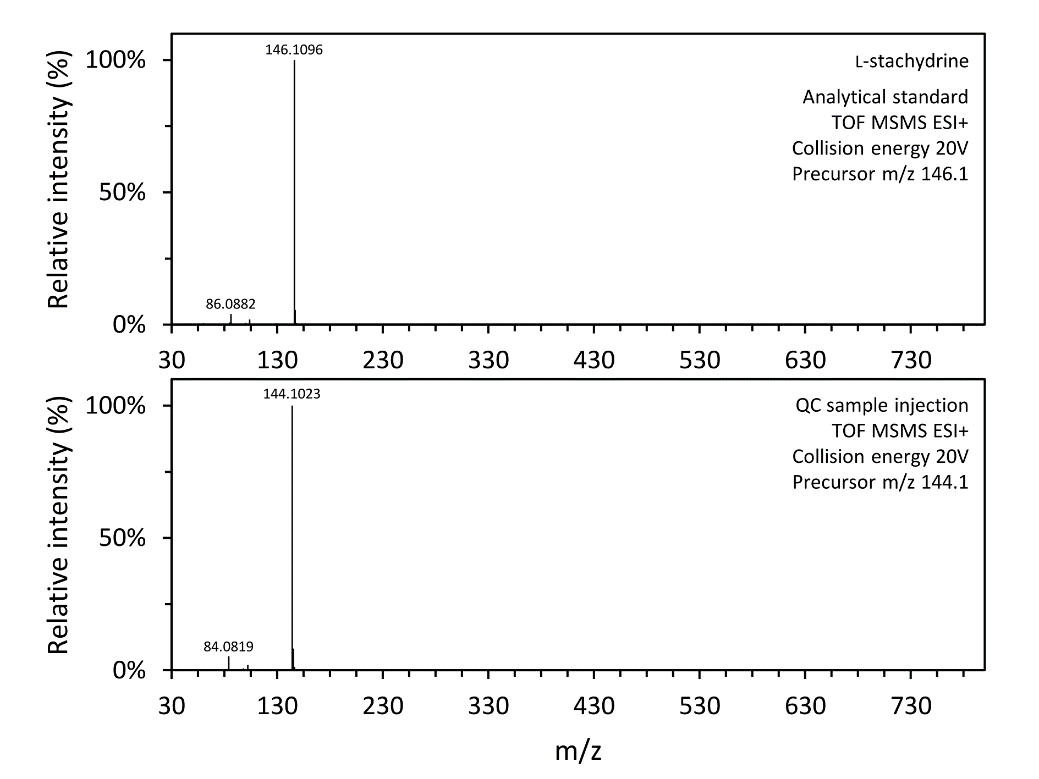


Figure S18 MS/MS spectrum of stachydrine. Collision energy 20V. Upper: analysis of analytical standard (13C2)-stachydrine. Precursor ion m/z 146.1. Lower: QC sample injection. Precursor ion 144.1.


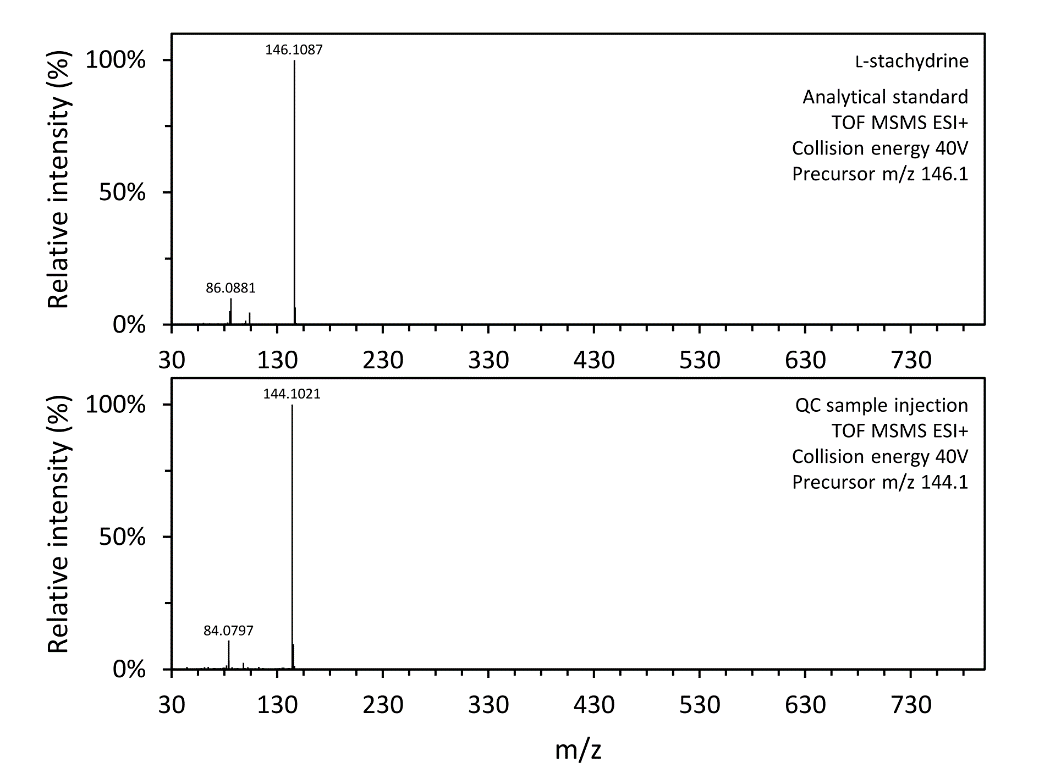


Figure S19 MS/MS spectrum of stachydrine. Collision energy 40V. Upper: analysis of analytical standard (13C2)-stachydrine. Precursor ion m/z 146.1. Lower: QC sample injection. Precursor ion 144.1.
